# Phase Two Steroid Metabolism and Its Roles in Breast and Prostate Cancer Patients

**DOI:** 10.3389/fendo.2013.00116

**Published:** 2013-09-04

**Authors:** Keely M. McNamara, Yasuhiro Nakamura, Yasuhiro Miki, Hironobu Sasano

**Affiliations:** ^1^Department of Pathology, Tohoku University School of Medicine, Miyagi, Sendai, Japan

**Keywords:** breast, prostate, intracrinology, STS inhibition, cancer

## Abstract

Breast and prostate cancer are diseases in which steroids and steroid metabolism could markedly influence clinical outcomes for patients. In both malignancies the modification of ketone and hydroxyl groups attached to the steroid backbone (phase one metabolism) has been examined in detail but the conjugation reactions (phase two metabolism) have not been extensively studied. Therefore, in this review we aim to summarize phase two metabolism in breast and prostate cancers from a number of perspectives, including the impact of variation in serum levels of conjugated steroids, tissue, and pathology specific expression of phase two enzymes, and consequences of genetic variations of these conjugation enzymes. In addition to this biological perspective, we will also address current pharmacological efforts to manipulate phase two metabolism as a potential therapy for hormone dependent cancers, including clinical trials of STS inhibitors and preclinical STS inhibitor development. While this review is not intended to cover any one particular area in great technical depth, it is intended as an introduction to and/or update on the importance of variance in phase two metabolic pathways in breast and prostate cancers.

## Introduction

Breast and prostate cancers are often characterized by their sex steroid dependence (1). A common characteristic of both malignancies is that steroidogenic enzymes and receptors have been reported as displaying prognostic significance (2, 3) and therefore the manipulation of steroidogenic pathways is a common mode of endocrine therapy in these cancers (4, 5). However, despite the importance of net steroid signaling to both cancers, the effects of variation in phase two metabolism have not necessarily been studied as much as those of phase one metabolism.

Phase two metabolism of androgens and estrogens is well known to be composed of two reversible conjugation pathways. In contrast to phase one metabolism, in which steroid potency is modulated through modifications between hydroxyl and ketone groups at the 3 and 17 carbon positions of the four carbon ring backbone structure common to all steroids, phase two metabolism is associated with the conjugation of a charged moiety at the three carbon position and is almost universally associated with a decrease in steroid potency (6). In the case of androgens and estrogens, this moiety corresponds to a sulfate or glucuronide group, giving rise to the naming of the two principle pathways of phase two metabolism in breast and prostate cancers; sulfation and glucuronidation.

**Figure 1:**
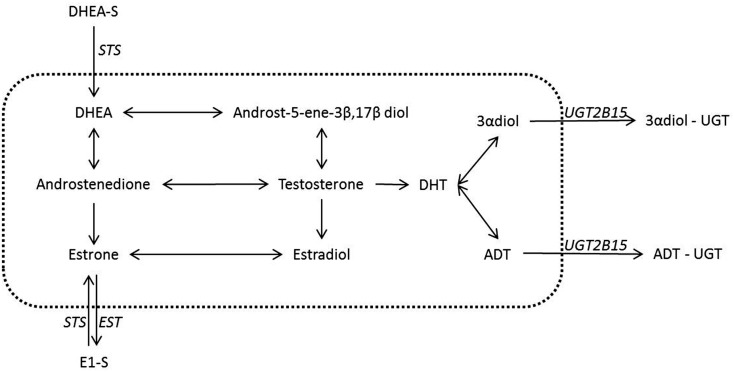
**Steroid conjugation reactions in breast and prostate with a known underlying biology**. Despite the many possible actions of steroids conjugation in the inactivation of steroids only a few have a concrete suggested role and have been well documented in the breast and the prostate. It is these latter reactions that are shown in the figure above. The reactions contained inside the dotted line are not conjugations reactions and hence are outside the scope of this review. These reactions however have been covered in recent reviews and we refer the reader toward these (1–3, 10). Italics indicate the known or most likely candidate enzymes that catalyze conjugation reactions. The arrows both in the central area and for the conjugation reactions give the possible directions for possible conversions, but in any tissue or state the nature of the conversions are dependent on the enzymes expressed.

The addition of sulfated and glucuronidated moieties to androgens and estrogens has the net effect of lowering receptor activation through both decreased potency and increased excretion (7–9). The reversibility of these reactions also means that conjugated steroids remain available, either from circulation or from local pools of steroids, to any tissues with the ability to de-conjugate the functional groups from the steroid backbone [e.g., Ref. (10)]. This latter point is important as it has been proposed that the secretion of conjugated and therefore inert steroids into the bloodstream, in tandem with tissue specific expression of steroid metabolizing enzymes, may allow tissue and/or organ specific steroid profiles to be created from a common circulating pool of steroids. Therefore, given the importance of both conjugation and de-conjugation reactions of androgens and estrogens in peripheral tissues, this review will focus on these pathways. These consist of four different reactions involving two functional groups – the conjugation and de-conjugation of glucuronide moieties to androgenic (C19) steroids and conjugation and the de-conjugation of sulfate moieties to either androgenic (C19) or estrogenic (C18/C19) steroids (Figure 1).

Sulfation in humans is, in general, either associated with the C19 precursor DHEA, estrone, or estradiol. The enzymes responsible for the sulfation of estradiol (E2) and estrone (E1) are SULT1A1, SULT1E1, and SULT2A1 (11), with each enzyme exhibiting a similar affinity for E2 or E1. Of these enzymes SULT1E1, also termed estrogen sulfotransferase (EST), is considered the principle C18 sulfation enzyme (12–14). Two different STS enzymes have been proposed in the reaction of C19 steroids, one which overlaps with estrogen metabolizing SULT enzymes [SULT2A1 (15)] while the other does not [SULT2B1 (16, 17)].

**Figure 2:**
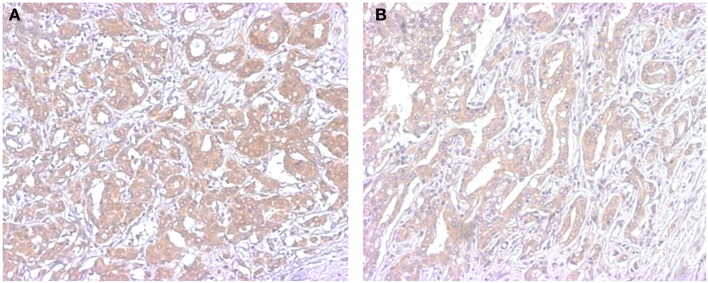
**Representative illustrations of immunohistochemistry of STS in breast (A) and prostate cancer (B)**. Immunoreactivity is detected in the cytoplasm of carcinoma cells.

In humans the enzyme responsible for de-conjugation of sulfated groups is common to both androgenic and estrogenic steroids. This enzyme is steroid sulfotransferase (STS), also termed aryl sulfotransferase. The relatively high levels of sulfated steroids in the circulation have made this particular enzyme the focus of intense study. These sulfated steroids are proposed to act as a pool of precursor steroids in tissues, such as the breast and the prostate, capable of deconjugating the sulfate moiety from the steroid backbone (Figure 2). This STS mediated enzymatic reaction is one of the most clinically relevant, at least within the confines of phase two metabolism, as it is the closest to being exploited as a therapeutic option in hormone dependent cancers.

Glucuronidated steroids, in contrast to sulfated steroids, have mostly been studied in the context of downstream metabolism of active androgens (Figure 1). Glucuronidated steroids derived from the potent androgen DHT have been reported to be measurable in the serum of both men (18, 19) and women (20, 21). The enzymes responsible for glucuronidation of androgens are UGT2B7, UGT2B15, UGT2B17, and possibly UGT2B28 (22), with recent studies suggesting specificity of different enzymes to steroids (23). While estrogen glucuronidation has not been extensively studied as a pathway in either breast or prostate cancer, activities of glucuronidation enzymes on estrogenic compounds have been reported and differ between estradiol and estrone (E2; UGT1A1, UGT1A4, UGT1A9, and UGT1A10 E1; UGT1A9, UGT1A10, UGT1A3, and UGT1A8) (11, 24). The glucuronidation of both C18 and C19 steroids is reversible and is mediated by β-Glucuronidase but this reverse reaction has not necessarily been well studied. This could be due to the relatively low levels of circulating glucuronidated androgens, suggesting that the de-conjugation of glucuronidated steroids may not play important roles in steroid dependent tissues.

## Conjugated Circulating Steroids and Hormone Dependent Cancer Risk

There is a relative abundance of conjugated as compared to non-conjugated steroids in the circulation [e.g., Ref. (25–27)], and variations in the levels of these steroids have been correlated with the risks of developing hormone dependent disease. In breast cancer increased serum concentrations of sulfated forms of both androgens and estrogens, along with their unconjugated forms, have been reported to be associated with increased risks of cancer development in some, but not all, studies of post-menopausal women (26, 28–31). The same possibility has been explored in prostate cancer but no association was demonstrated (32).

Associations between serum glucuronidated steroid levels and risks of hormonal dependent cancer development have been principally studied in prostate cancer and, in this setting, glucuronidated steroid have been interpreted as indirect markers of peripheral androgen synthesis (33). For instance in Japanese compared to western males, the main circulating glucuronidated steroids androsterone glucuronide (ADT-G) and androstane-3α, 17β-diol glucuronide (3α-diol-G) are lower (34). This has been interpreted to suggest that lower peripheral steroid synthesis could account for the lower prostate cancer risk in Japanese males (34). Measurable levels of serum glucuronidated androgens have also been reported in females (20, 21) but their clinical significance remains unknown with regard to the risk of breast cancer development.

## Steroid Conjugating Enzymes in Breast and Prostate Tissues

While serum levels of conjugated steroids provide information as to the sum total of conjugation reaction in the entire body they do not necessarily provide information regarding the steroid metabolism within a particular tissue. Hence an examination of the localized expression of relevant enzymes is considered invaluable in demonstrating the role(s) of phase two metabolism specific to a tissue and its pathology. Such studies have mainly focused on EST expression, principally in breast cancer patients but also to a limited extent in prostate cancer patients.

In the prostate, EST immunoreactivity was reported to be absent in normal ductal cells but detected in carcinoma cells (35) suggesting a possible role in cancer. In contrast to prostate cancer, the roles of steroid sulfating enzymes in breast cancer, especially that of EST, have been extensively studied. In human breast tissue EST was detected in both normal and various carcinoma tissues (36–39). EST expression is generally most pronounced in the normal breast followed by ductal carcinoma *in situ* (DCIS) and lowest in invasive ductal carcinoma with localization to both the tumor and tumor-adjacent stromal fibroblasts (36–38). An inverse correlation between tumor histological grade and the levels of intratumoral EST immunoreactivity was also reported in both invasive carcinoma and DCIS (38, 39). This suggests that the inactivation of estrogens by EST is an important component in protecting the breast against estrogen excess, thus averting malignant growth. In addition to EST, SULT2B1 expression was present in both breast and prostate cancers (16, 17) and in the breast its expression was reported to be increased in cancerous as compared to normal breast tissues (16, 40). This finding suggests a potential role for androgens in protecting breast tissues. However, further investigations are needed to confirm or disprove this potentially interesting hypothesis.

Glucuronidation enzymes in the breast [C18; UGT1A1, UGT1A8, and UGT1A10, UGT2B28 (24, 41, 42) C19; UGT2B15 and UGT2B28 (24, 41)] and prostate [C18; UGT1A5, UGT1A10 and UGT1A1 (24, 42) C19; UGT2B15 and UGT2B17 (24)] have been reported but their precise clinical and/or biological significance is not clear. One recent study has examined the localization of UGT2B15 and UGT2B17 in prostate cancer showing that UGT2B17 increased, and UGT2B15 decreased in cancer progression from benign disease to lymph node metastasis (43). Further investigations such as these in breast and prostate cancer may bring new and interesting insights into the underlying biology.

## Genetic Polymorphisms in Conjugating Enzymes and Breast and Prostate Cancer Risk

For a number of the enzymes detailed above there are validated polymorphisms reported with characterized alterations in enzyme activity. When probing the relevance of these polymorphisms in breast and prostate cancer two potential roles have been identified; the impact of polymorphisms upon the tissue metabolism of endogenous steroids and the potential impact of the polymorphisms upon the metabolism of chemical or endocrine directed therapy. The latter is considered important but it is outside the scope of this review and we direct the reader toward original papers dealing with this topic (44–46). In this section we will concentrate on the potential impact of polymorphisms in treatment-naïve settings.

In breast cancer, studies have focused on polymorphisms in UGT1A1, SULTE1/EST, and SULT1A1. For UGT1A1, the majority of studies examining genetic variation have evaluated the impact of variants associated with a lower rate of enzyme transcription *in vitro*. These studies all demonstrated an association between the low activity variant and an increased risk of premenopausal breast cancer across a number of ethnic populations [African (47), Chinese (48), African Americans (49)] and in addition an association with cancer grade, estrogen negativity and increased mammographic density (50, 51). A similar association between genetic variants in SULT1E1 and breast cancer risk/mammographic density has also been detected (51–53). These findings are not incontrovertible as in other studies no association between clinical factors and SNPs in SULT1E1 was reported (54).

In prostate cancer studies have focused the UGT2B15(D85Y) and the UGT2B17 gene deletion variants, although a correlation between variations in the gene copy number and serum steroids was also reported (55, 56). In men, UGT2B15(D85Y) and the UGT2B17 deletion were both associated with alterations in serum steroid levels and fat mass (23, 57, 58) and the UGT215(D85Y) variation (56, 59) has been reported to be associated with increased prostate cancer risk in unselected (59), Caucasian (60) and Japanese (61) subjects. However, it is also true that other studies failed to detect an association between increased prostate cancer incidence and UGT2B17 gene deletion variation in Caucasian (62) and African American (60) patients.

Given that the polymorphisms described above have been shown to alter serum levels of steroids, it is possibly unsurprising that they are also associated with increases in hormone dependent cancer risk. While this data does not specifically show that enzyme alterations in the breast or prostate tissue leads to malignancy it does serve as a useful illustration of the importance of steroid conjugating enzymes in protecting tissues against steroid excess, and the consequences to the organism when this system is disrupted. Variations in findings among reported studies may be attributable to multiple factors (including study size and power) and require further investigation to fully unravel any associations.

## Hormonal Regulation as a Modulator of Phase Two Metabolism in Breast and Prostate Cancer

Hormonal regulation of the inactivation of steroids is important because the endocrine treatment for both breast and prostate cancer involves the manipulation of various hormonal signaling pathways. Additionally in breast cancer, the presence or absence of various sex steroid receptors subdivides the disease into clinically meaningful subgroups with unique treatment strategies and different prognosis. These suggest that an understanding of the regulation of phase two metabolism by hormones may be helpful in understanding and potentially manipulating it in breast and prostate cancer.

The hormonal regulation of phase two metabolism has not been reported in the peripheral tissues with the exception of EST in the endometrium (63, 64). EST expression in the endometrium was reported to be hormone dependent as it paralleled the serum progesterone levels during the menstrual cycle and was suppressed by oral contraceptives (64). While *in vivo* studies in breast cancer have not been reported in the literature, *in vitro* studies of breast cancer cell lines have demonstrated estrogen treatment and increases in cell density respectively up- and down-regulate EST expression (63, 65). Additional *in vitro* studies have demonstrated androgen dependent down-regulation and estrogen dependent up-regulation of androgenic and estrogenic UGT subtypes in breast and prostate carcinoma cell lines respectively (24). Although preliminary, these findings may indicate that the local microenvironment including intratumoral levels of steroid impact phase two metabolism which may in turn influence the levels of available steroids and contribute to disease progression.

## Manipulation of De-Conjugation Enzymes in the Treatment of Breast and Prostate Cancer

In breast cancer patients, estrogenic signaling is the best characterized driver of carcinoma cell proliferation and therefore much research has been devoted to how to reduce the levels of estrogenic signaling in carcinoma cells. Current first line therapy in estrogen dependent post-menopausal breast cancer patients usually employs aromatase inhibitors to suppress the production of intratumoral estrogens from androgenic precursors. This approach could eliminate one source of estrogens but the second potential source for estrogenic signaling, the conversion of sulfated estrogens to un-sulfated estrogens by STS, still remain. The adverse roles of STS in breast cancer have been proposed by several previous studies. STS expression was reported to be increased in female breast cancer patients (10, 66) and increased expression has also been associated with increased recurrence (67), clinical resistance to endocrine therapies (68) and higher histological grades (10, 69). Interestingly this pathway may not be as active in male breast cancer patients (70), suggesting a gender difference in the intracrine metabolism of the breast. Additionally while the roles of STS in the generation of estrogenic signal have been well studied, its potential roles of generating intratumoral androgens from DHEA-S are not, despite the growing awareness of the potential impact of androgen metabolism in breast cancers (71–73). The potential for STS to act as a source of both estrogenic and androgenic precursors in prostate cancer is similar to breast cancer, but this field has been less explored in the prostate. The activity of STS has been studied in human prostate carcinoma cell lines and its immunoreactivity was detected in carcinoma tissues (35), which is consistent with the potential of this mode of therapy.

The road to developing inhibitors capable of targeting STS has been a long one involving multiple iterations of steroidal and non-steroidal compounds over the last three decades. As this has been comprehensively covered in a number of recent reviews [e.g., Ref. (10, 74, 75)], in the interests of space we will focus on the latest developments regarding inhibitor design and the current state of clinical trials of STS inhibitors.

Initial efforts in developing compounds to inhibit STS activity uncovered the potential of the sulfamate group as an irreversible inhibitor of STS. The addition of this functional group to an estrogenic steroid backbone led to the first promising irreversible STS inhibitor estrone-3-*O*-sulfamate (EMATE) (76). Despite positive *in vitro* data EMATE demonstrated estrogenic properties in rodents (77) which, given its proposed application in estrogen responsive cancers, diminished its usefulness as a human therapeutic agent. As the estrogenicity of EMATE was attributed to the release of the steroid backbone during inhibition (77), subsequent iterations of STS inhibitor design have focused upon two main strategies to overcome this drawback. Both of these approaches have focused on changing the molecule to which sulfamate groups are bound while still relying on phenol sulfamate ester pharmacophores for enzyme inhibition.

The first approach focused on the use of non-steroidal structures as compounds to hold the sulfamate functional group. This approach has resulted in the development of STX64 (667COUMATE, BN83495, irosustat), the only STS inhibitor to date to reach phase two clinical trials. Initial phase one trials in breast cancer patients were considered promising with an observation of stable disease in the trial participants (78–80). However, in 2011, futility analysis of a phase II trial of STX64 following chemotherapy in ER+ post-menopausal endometrial cancer (NCT01251354) suggested no effect and led to the discontinuation of this trial, and a discontinuation of Ipsen sponsored development of STX64 (81). This decision which impacted a phase II trial of STX64 as a therapeutic agent in post-menopausal treatment-naïve breast cancer (NCT01230970) and a phase one trial of STX64 in metastatic prostate cancer (NCT00790374). Despite this setback, other phase II trials addressing the possibility of STX64 as a combination therapy in advanced ER+ breast cancer (NCT01785992) and the potential of STX64 as a preoperative treatment in treatment-naïve breast cancer (NCT01662726) are currently recruiting. In addition to alternative uses of STX64, other structures with a similar design strategy have been patented and are in development (75).

The second approach to avoiding unwanted estrogenicity in STS inhibitors has focused on modifications to the steroid back bone to render it non-estrogenic. Compounds of this nature have been patented (75). These have shown *in vitro* potency and a lack of estrogenicity (75, 82, 83) with proven efficacy in rodent models (75, 84–86). However, to date, none have progressed to being tested in human clinical trials. This approach remains an exciting prospect for the future.

Not mentioned above are compound STS inhibitors which, while not having yet reached clinical testing, are an area of active development. Compound inhibitors aim to utilize the cleavage of the sulfamate group from the parental compound as a means to deliver two drugs in one by utilizing a biologically active parental compound. Concepts utilizing this strategy have principally focused on potential uses in breast cancer and thus encompass dual STS/SERMs, STS/aromatase, and STS/17βHSD1 inhibitors, recently reviewed in Ref. (75). Among these three different groups, the most advanced along the development pipeline is that of STS/aromatase dual inhibitors with promising results in rodent models (87).

## Summary

The targeting of phase two metabolism in breast and prostate cancer is considered a promising emergent therapy. Most of the research has focused on STS, and specific inhibition of this enzyme could become an effective therapeutic tool in estrogen dependent breast cancer and possibly in prostate cancer patients. However, more research on the role of phase two metabolism in the excretion of androgens and estrogens is warranted to fully understand its significance in breast and prostate cancers.

## Conflict of Interest Statement

The authors declare that the research was conducted in the absence of any commercial or financial relationships that could be construed as a potential conflict of interest.

## References

[1] Risbridger GP Davis ID Birrell SN Tilley WD Breast and prostate cancer: more similar than different. Nat Rev Cancer (2010) 10(3):205–12 10.1038/nrc2795 20147902

[2] Sharifi N Auchus RJ Steroid biosynthesis and prostate cancer. Steroids (2012) 77(7):719–26 10.1016/j.steroids.2012.03.015 22503713

[3] Sasano H Suzuki T Miki Y Moriya T Intracrinology of estrogens and androgens in breast carcinoma. J Steroid Biochem Mol Biol (2008) 108(3–5):181–5 10.1016/j.jsbmb.2007.09.012 17933521

[4] Montorsi F Alcaraz A Desgrandchamps F Hammerer P Schroder F Castro R A broader role for 5ARIs in prostate disease? Existing evidence and emerging benefits. Prostate (2009) 69(8):895–907 10.1002/pros.20939 19267353

[5] Santen RJ Brodie H Simpson ER Siiteri PK Brodie A History of aromatase: saga of an important biological mediator and therapeutic target. Endocr Rev (2009) 30(4):343–75 10.1210/er.2008-0016 19389994

[6] Gosetti F Mazzucco E Gennaro MC Marengo E Ultra high performance liquid chromatography tandem mass spectrometry determination and profiling of prohibited steroids in human biological matrices. A review. J Chromatogr B Analyt Technol Biomed Life Sci (2013) 927:22–36 10.1016/j.jchromb.2012.12.003 23317577

[7] Nozaki O Steroid analysis for medical diagnosis. J Chromatogr A (2001) 935(1–2):267–78 10.1016/S0021-9673(01)01104-9 11762779

[8] Klyne W Schachter B Marrian GF The steroids of pregnant mares’ urine. 1. A method for the extraction of steroid sulphates and the isolation of allopregn-16-en-3(beta)-ol-20-one sulphate. Biochem J (1948) 43(2):231–4 PMC1274672 16748393

[9] Barry MC Eidinoff ML Dobriner K Gallagher TF The fate of C14 testosterone and C14-progesterone in mice and rats. Endocrinology (1952) 50(6):587–99 10.1210/endo-50-6-587 12980068

[10] Geisler J Sasano H Chen S Purohit A Steroid sulfatase inhibitors: promising new tools for breast cancer therapy? J Steroid Biochem Mol Biol (2011) 125(1–2):39–45 10.1016/j.jsbmb.2011.02.002 21356310

[11] Raftogianis R Creveling C Weinshilboum R Weisz J Estrogen metabolism by conjugation. J Natl Cancer Inst Monogr (2000) 27:113–24 10.1093/oxfordjournals.jncimonographs.a024234 10963623

[12] Pasqualini JR Estrogen sulfotransferases in breast and endometrial cancers. Ann N Y Acad Sci (2009) 1155:88–98 10.1111/j.1749-6632.2009.04113.x 19250196

[13] Xu Y Liu X Guo F Ning Y Zhi X Wang X Effect of estrogen sulfation by SULT1E1 and PAPSS on the development of estrogen-dependent cancers. Cancer Sci (2012) 103(6):1000–9 10.1111/j.1349-7006.2012.02258.x 22380844 PMC7685083

[14] Zhang H Varlamova O Vargas FM Falany CN Leyh TS Sulfuryl transfer: the catalytic mechanism of human estrogen sulfotransferase. J Biol Chem (1998) 273(18):10888–92 10.1074/jbc.273.18.10888 9556564

[15] Cook I Wang T Almo SC Kim J Falany CN Leyh TS Testing the sulfotransferase molecular pore hypothesis. J Biol Chem (2013) 288(12):8619–26 10.1074/jbc.M112.445015 23362278 PMC3605680

[16] Falany CN He D Dumas N Frost AR Falany JL Human cytosolic sulfotransferase 2B1: isoform expression, tissue specificity and subcellular localization. J Steroid Biochem Mol Biol (2006) 102(1–5):214–21 10.1016/j.jsbmb.2006.09.011 17055258 PMC1820847

[17] Geese WJ Raftogianis RB Biochemical characterization and tissue distribution of human SULT2B1. Biochem Biophys Res Commun (2001) 288(1):280–9 10.1006/bbrc.2001.5746 11594786

[18] Belanger A Hum DW Beaulieu M Levesque E Guillemette C Tchernof A Characterization and regulation of UDP-glucuronosyltransferases in steroid target tissues. J Steroid Biochem Mol Biol (1998) 65(1–6):301–10 10.1016/S0960-0760(97)00183-0 9699884

[19] Moghissi E Ablan F Horton R Origin of plasma androstanediol glucuronide in men. J Clin Endocrinol Metab (1984) 59(3):417–21 10.1210/jcem-59-3-417 6746859

[20] Falsetti L Rosina B De Fusco D Serum levels of 3alpha-androstanediol glucuronide in hirsute and non hirsute women. Eur J Endocrinol (1998) 138(4):421–4 10.1530/eje.0.1380421 9578510

[21] Rittmaster RS Thompson DL Listwak S Loriaux DL Androstanediol glucuronide isomers in normal men and women and in men infused with labeled dihydrotestosterone. J Clin Endocrinol Metab (1988) 66(1):212–6 10.1210/jcem-66-1-212 3335605

[22] Barbier O Belanger A Inactivation of androgens by UDP-glucuronosyltransferases in the human prostate. Best Pract Res Clin Endocrinol Metab (2008) 22(2):259–70 10.1016/j.beem.2008.01.001 18471784

[23] Swanson C Mellstrom D Lorentzon M Vandenput L Jakobsson J Rane A The uridine diphosphate glucuronosyltransferase 2B15 D85Y and 2B17 deletion polymorphisms predict the glucuronidation pattern of androgens and fat mass in men. J Clin Endocrinol Metab (2007) 92(12):4878–82 10.1210/jc.2007-0359 17698910

[24] Mackenzie PI Hu DG Gardner-Stephen DA The regulation of UDP-glucuronosyltransferase genes by tissue-specific and ligand-activated transcription factors. Drug Metab Rev (2010) 42(1):99–109 10.3109/03602530903209544 20070244

[25] Hankinson SE Willett WC Manson JE Colditz GA Hunter DJ Spiegelman D Plasma sex steroid hormone levels and risk of breast cancer in postmenopausal women. J Natl Cancer Inst (1998) 90(17):1292–9 10.1093/jnci/90.17.1292 9731736

[26] Pasqualini JR Chetrite G Blacker C Feinstein MC Delalonde L Talbi M Concentrations of estrone, estradiol, and estrone sulfate and evaluation of sulfatase and aromatase activities in pre- and postmenopausal breast cancer patients. J Clin Endocrinol Metab (1996) 81(4):1460–4 10.1210/jc.81.4.1460 8636351

[27] Chouinard S Yueh MF Tukey RH Giton F Fiet J Pelletier G Inactivation by UDP-glucuronosyltransferase enzymes: the end of androgen signaling. J Steroid Biochem Mol Biol (2008) 109(3–5):247–53 10.1016/j.jsbmb.2008.03.016 18467088

[28] Zeleniuch-Jacquotte A Shore RE Koenig KL Akhmedkhanov A Afanasyeva Y Kato I Postmenopausal levels of oestrogen, androgen, and SHBG and breast cancer: long-term results of a prospective study. Br J Cancer (2004) 90(1):153–9 10.1038/sj.bjc.6601517 14710223 PMC2395327

[29] Kaaks R Rinaldi S Key TJ Berrino F Peeters PH Biessy C Postmenopausal serum androgens, oestrogens and breast cancer risk: the European prospective investigation into cancer and nutrition. Endocr Relat Cancer (2005) 12(4):1071–82 10.1677/erc.1.01038 16322344

[30] Missmer SA Eliassen AH Barbieri RL Hankinson SE Endogenous estrogen, androgen, and progesterone concentrations and breast cancer risk among postmenopausal women. J Natl Cancer Inst (2004) 96(24):1856–65 10.1093/jnci/djh336 15601642

[31] Key T Appleby P Barnes I Reeves G Endogenous sex hormones and breast cancer in postmenopausal women: reanalysis of nine prospective studies. J Natl Cancer Inst (2002) 94(8):606–16 10.1093/jnci/94.8.606 11959894

[32] Roddam AW Allen NE Appleby P Key TJ Endogenous sex hormones and prostate cancer: a collaborative analysis of 18 prospective studies. J Natl Cancer Inst (2008) 100(3):170–83 10.1093/jnci/djm323 18230794 PMC6126902

[33] Belanger A Brochu M Cliche J Levels of plasma steroid glucuronides in intact and castrated men with prostatic cancer. J Clin Endocrinol Metab (1986) 62(5):812–5 10.1210/jcem-62-5-812 2937801

[34] Ross RK Bernstein L Lobo RA Shimizu H Stanczyk FZ Pike MC 5-alpha-reductase activity and risk of prostate cancer among Japanese and US white and black males. Lancet (1992) 339(8798):887–9 10.1016/0140-6736(92)90927-U 1348296

[35] Nakamura Y Suzuki T Fukuda T Ito A Endo M Moriya T Steroid sulfatase and estrogen sulfotransferase in human prostate cancer. Prostate (2006) 66(9):1005–12 10.1002/pros.20426 16541422

[36] Suzuki T Nakata T Miki Y Kaneko C Moriya T Ishida T Estrogen sulfotransferase and steroid sulfatase in human breast carcinoma. Cancer Res (2003) 63(11):2762–70 12782580

[37] Suzuki M Ishida H Shiotsu Y Nakata T Akinaga S Takashima S Expression level of enzymes related to in situ estrogen synthesis and clinicopathological parameters in breast cancer patients. J Steroid Biochem Mol Biol (2009) 113(3–5):195–201 10.1016/j.jsbmb.2008.12.008 19159687

[38] Hudelist G Wulfing P Kersting C Burger H Mattsson B Czerwenka K Expression of aromatase and estrogen sulfotransferase in preinvasive and invasive breast cancer. J Cancer Res Clin Oncol (2008) 134(1):67–73 10.1007/s00432-007-0249-2 17661084 PMC12161745

[39] Suzuki T Miki Y Nakamura Y Ito K Sasano H Steroid sulfatase and estrogen sulfotransferase in human carcinomas. Mol Cell Endocrinol (2011) 340(2):148–53 10.1016/j.mce.2010.11.001 21073915

[40] Bieche I Girault I Urbain E Tozlu S Lidereau R Relationship between intratumoral expression of genes coding for xenobiotic-metabolizing enzymes and benefit from adjuvant tamoxifen in estrogen receptor alpha-positive postmenopausal breast carcinoma. Breast Cancer Res (2004) 6(3):R252–63 10.1186/bcr784 15084249 PMC400681

[41] Belanger A Pelletier G Labrie F Barbier O Chouinard S Inactivation of androgens by UDP-glucuronosyltransferase enzymes in humans. Trends Endocrinol Metab (2003) 14(10):473–9 10.1016/j.tem.2003.10.005 14643063

[42] Ohno S Nakajin S Determination of mRNA expression of human UDP-glucuronosyltransferases and application for localization in various human tissues by real-time reverse transcriptase-polymerase chain reaction. Drug Metab Dispos (2009) 37(1):32–40 10.1124/dmd.108.023598 18838504

[43] Paquet S Fazli L Grosse L Verreault M Tetu B Rennie PS Differential expression of the androgen-conjugating UGT2B15 and UGT2B17 enzymes in prostate tumor cells during cancer progression. J Clin Endocrinol Metab (2012) 97(3):E428–32 10.1210/jc.2011-2064 22170718

[44] Nowell SA Ahn J Rae JM Scheys JO Trovato A Sweeney C Association of genetic variation in tamoxifen-metabolizing enzymes with overall survival and recurrence of disease in breast cancer patients. Breast Cancer Res Treat (2005) 91(3):249–58 10.1007/s10549-004-7751-x 15952058

[45] Wegman P Elingarami S Carstensen J Stal O Nordenskjold B Wingren S Genetic variants of CYP3A5, CYP2D6, SULT1A1, UGT2B15 and tamoxifen response in postmenopausal patients with breast cancer. Breast Cancer Res (2007) 9(1):R7 10.1186/bcr1640 17244352 PMC1851378

[46] Ahern TP Christensen M Cronin-Fenton DP Lunetta KL Soiland H Gjerde J Functional polymorphisms in UDP-glucuronosyl transferases and recurrence in tamoxifen-treated breast cancer survivors. Cancer Epidemiol Biomarkers Prev (2011) 20(9):1937–43 10.1158/1055-9965.EPI-11-0419 21750172 PMC3169710

[47] Huo D Kim HJ Adebamowo CA Ogundiran TO Akang EE Campbell O Genetic polymorphisms in uridine diphospho-glucuronosyltransferase 1A1 and breast cancer risk in Africans. Breast Cancer Res Treat (2008) 110(2):367–76 10.1007/s10549-007-9720-7 17909964 PMC4384416

[48] Adegoke OJ Shu XO Gao YT Cai Q Breyer J Smith J Genetic polymorphisms in uridine diphospho-glucuronosyltransferase 1A1 (UGT1A1) and risk of breast cancer. Breast Cancer Res Treat (2004) 85(3):239–45 10.1023/B:BREA.0000025419.26423.b8 15111762

[49] Guillemette C Millikan RC Newman B Housman DE Genetic polymorphisms in uridine diphospho-glucuronosyltransferase 1A1 and association with breast cancer among African Americans. Cancer Res (2000) 60(4):950–6 10706110

[50] Shatalova EG Walther SE Favorova OO Rebbeck TR Blanchard RL Genetic polymorphisms in human SULT1A1 and UGT1A1 genes associate with breast tumor characteristics: a case-series study. Breast Cancer Res (2005) 7(6):R909–21 10.1186/bcr1318 16280036 PMC1410736

[51] Yong M Schwartz SM Atkinson C Makar KW Thomas SS Newton KM Associations between polymorphisms in glucuronidation and sulfation enzymes and mammographic breast density in premenopausal women in the United States. Cancer Epidemiol Biomarkers Prev (2010) 19(2):537–46 10.1158/1055-9965.EPI-09-0898 20142249 PMC2820123

[52] Adjei AA Thomae BA Prondzinski JL Eckloff BW Wieben ED Weinshilboum RM Human estrogen sulfotransferase (SULT1E1) pharmacogenomics: gene resequencing and functional genomics. Br J Pharmacol (2003) 139(8):1373–82 10.1038/sj.bjp.0705369 12922923 PMC1573968

[53] Choi JY Lee KM Park SK Noh DY Ahn SH Chung HW Genetic polymorphisms of SULT1A1 and SULT1E1 and the risk and survival of breast cancer. Cancer Epidemiol Biomarkers Prev (2005) 14(5):1090–5 10.1158/1055-9965.EPI-04-0688 15894657

[54] Udler MS Azzato EM Healey CS Ahmed S Pooley KA Greenberg D Common germline polymorphisms in COMT, CYP19A1, ESR1, PGR, SULT1E1 and STS and survival after a diagnosis of breast cancer. Int J Cancer (2009) 125(11):2687–96 10.1002/ijc.24678 19551860

[55] Menard V Eap O Harvey M Guillemette C Levesque E Copy-number variations (CNVs) of the human sex steroid metabolizing genes UGT2B17 and UGT2B28 and their associations with a UGT2B15 functional polymorphism. Hum Mutat (2009) 30(9):1310–9 10.1002/humu.21054 19572376

[56] Grant DJ Hoyo C Oliver SD Gerber L Shuler K Calloway E Association of uridine diphosphate-glucuronosyltransferase 2B gene variants with serum glucuronide levels and prostate cancer risk. Genet Test Mol Biomarkers (2013) 17(1):3–9 10.1089/gtmb.2012.0161 23098242 PMC3525886

[57] Swanson C Lorentzon M Vandenput L Labrie F Rane A Jakobsson J Sex steroid levels and cortical bone size in young men are associated with a uridine diphosphate glucuronosyltransferase 2B7 polymorphism (H268Y). J Clin Endocrinol Metab (2007) 92(9):3697–704 10.1210/jc.2007-0530 17579197

[58] Olsson M Ekstrom L Guillemette C Belanger A Rane A Gustafsson O Correlation between circulatory, local prostatic, and intra-prostatic androgen levels. Prostate (2011) 71(9):909–14 10.1002/pros.21307 21541968

[59] Nadeau G Bellemare J Audet-Walsh E Flageole C Huang SP Bao BY Deletions of the androgen-metabolizing UGT2B genes have an effect on circulating steroid levels and biochemical recurrence after radical prostatectomy in localized prostate cancer. J Clin Endocrinol Metab (2011) 96(9):E1550–7 10.1210/jc.2011-1049 21733997

[60] Park J Chen L Ratnashinge L Sellers TA Tanner JP Lee JH Deletion polymorphism of UDP-glucuronosyltransferase 2B17 and risk of prostate cancer in African American and Caucasian men. Cancer Epidemiol Biomarkers Prev (2006) 15(8):1473–8 10.1158/1055-9965.EPI-06-0141 16896035

[61] Okugi H Nakazato H Matsui H Ohtake N Nakata S Suzuki K Association of the polymorphisms of genes involved in androgen metabolism and signaling pathways with familial prostate cancer risk in a Japanese population. Cancer Detect Prev (2006) 30(3):262–8 10.1016/j.cdp.2006.04.004 16859836

[62] Gallagher CJ Kadlubar FF Muscat JE Ambrosone CB Lang NP Lazarus P The UGT2B17 gene deletion polymorphism and risk of prostate cancer. A case-control study in Caucasians. Cancer Detect Prev (2007) 31(4):310–5 10.1016/j.cdp.2007.07.005 17935910 PMC2096411

[63] Coughtrie MW Sharp S Maxwell K Innes NP Biology and function of the reversible sulfation pathway catalysed by human sulfotransferases and sulfatases. Chem Biol Interact (1998) 109(1-3):3–27 10.1016/S0009-2797(97)00117-8 9566730

[64] Rubin GL Harrold AJ Mills JA Falany CN Coughtrie MW Regulation of sulphotransferase expression in the endometrium during the menstrual cycle, by oral contraceptives and during early pregnancy. Mol Hum Reprod (1999) 5(11):995–1002 10.1093/molehr/5.11.995 10541560

[65] Fu J Fang H Paulsen M Ljungman M Kocarek TA Runge-Morris M Regulation of estrogen sulfotransferase expression by confluence of MCF10A breast epithelial cells: role of the aryl hydrocarbon receptor. J Pharmacol Exp Ther (2011) 339(2):597–606 10.1124/jpet.111.185173 21828262 PMC3199978

[66] Suzuki T Miki Y Nakata T Shiotsu Y Akinaga S Inoue K Steroid sulfatase and estrogen sulfotransferase in normal human tissue and breast carcinoma. J Steroid Biochem Mol Biol (2003) 86(3–5):449–54 10.1016/S0960-0760(03)00356-X 14623543

[67] Utsumi T Yoshimura N Takeuchi S Ando J Maruta M Maeda K Steroid sulfatase expression is an independent predictor of recurrence in human breast cancer. Cancer Res (1999) 59(2):377–81 9927050

[68] Chanplakorn N Chanplakorn P Suzuki T Ono K Chan MS Miki Y Increased estrogen sulfatase (STS) and 17beta-hydroxysteroid dehydrogenase type 1(17beta-HSD1) following neoadjuvant aromatase inhibitor therapy in breast cancer patients. Breast Cancer Res Treat (2010) 120(3):639–48 10.1007/s10549-010-0785-3 20151319

[69] Al Sarakbi W Mokbel R Salhab M Jiang WG Reed MJ Mokbel K The role of STS and OATP-B mRNA expression in predicting the clinical outcome in human breast cancer. Anticancer Res (2006) 26(6C):4985–90 17214375

[70] Takagi K Moriya T Kurosumi M Oka K Miki Y Ebata A Intratumoral estrogen concentration and expression of estrogen-induced genes in male breast carcinoma: comparison with female breast carcinoma. Horm Cancer (2013) 4(1):1–11 10.1007/s12672-012-0126-6 23096432 PMC10357998

[71] Hickey TE Robinson JL Carroll JS Tilley WD Minireview: the androgen receptor in breast tissues: growth inhibitor, tumor suppressor, oncogene? Mol Endocrinol (2012) 26(8):1252–67 10.1210/me.2012-1107 22745190 PMC3404296

[72] McNamara KM Yoda T Takagi K Miki Y Suzuki T Sasano H Androgen receptor in triple negative breast cancer. J Steroid Biochem Mol Biol (2013) 133:66–76 10.1016/j.jsbmb.2012.08.007 22982153

[73] Suzuki T Miki Y Moriya T Akahira J Ishida T Hirakawa H 5α-reductase type 1 and aromatase in breast carcinoma as regulators of in situ androgen production. Int J Cancer (2007) 120(2):285–91 10.1002/ijc.22317 17066438

[74] Purohit A Foster PA Steroid sulfatase inhibitors for estrogen- and androgen-dependent cancers. J Endocrinol (2012) 212(2):99–110 10.1530/JOE-11-0266 21859802

[75] Williams SJ Sulfatase inhibitors: a patent review. Expert Opin Ther Pat (2013) 23(1):79–98 10.1517/13543776.2013.736965 23136854

[76] Purohit A Williams GJ Roberts CJ Potter BV Reed MJ In vivo inhibition of oestrone sulphatase and dehydroepiandrosterone sulphatase by oestrone-3-O-sulphamate. Int J Cancer (1995) 63(1):106–11 10.1002/ijc.2910630119 7558436

[77] Elger W Schwarz S Hedden A Reddersen G Schneider B Sulfamates of various estrogens are prodrugs with increased systemic and reduced hepatic estrogenicity at oral application. J Steroid Biochem Mol Biol (1995) 55(3–4):395–403 10.1016/0960-0760(95)00214-6 8541236

[78] Stanway SJ Purohit A Woo LW Sufi S Vigushin D Ward R Phase I study of STX 64 (667 Coumate) in breast cancer patients: the first study of a steroid sulfatase inhibitor. Clin Cancer Res (2006) 12(5):1585–92 10.1158/1078-0432.CCR-05-1996 16533785

[79] Coombes RC Cardoso F Isambert N Lesimple T Soulie P Peraire C A phase I dose escalation study to determine the optimal biological dose of irosustat, an oral steroid sulfatase inhibitor, in postmenopausal women with estrogen receptor-positive breast cancer. Breast Cancer Res Treat (2013) 140(1):73–82 10.1007/s10549-013-2597-8 23797179

[80] Mostafa YA Taylor SD Steroid derivatives as inhibitors of steroid sulfatase. J Steroid Biochem Mol Biol (2013). 10.1016/j.jsbmb.2013.01.013 23391659

[81] Ipsen Results and 2013 Financial Objectives 2012 (2012). Available from: http://www.ipsen.com/en/ipsens-2012-results-and-2013-financial-objectives

[82] Fischer DS Chander SK Woo LW Fenton JC Purohit A Reed MJ Novel D-ring modified steroid derivatives as potent, non-estrogenic, steroid sulfatase inhibitors with in vivo activity. J Steroid Biochem Mol Biol (2003) 84(2–3):343–9 10.1016/S0960-0760(03)00048-7 12711021

[83] Woo LW Fischer DS Sharland CM Trusselle M Foster PA Chander SK Anticancer steroid sulfatase inhibitors: synthesis of a potent fluorinated second-generation agent, in vitro and in vivo activities, molecular modeling, and protein crystallography. Mol Cancer Ther (2008) 7(8):2435–44 10.1158/1535-7163.MCT-08-0195 18723489

[84] Foster PA Newman SP Chander SK Stengel C Jhalli R Woo LL In vivo efficacy of STX213, a second-generation steroid sulfatase inhibitor, for hormone-dependent breast cancer therapy. Clin Cancer Res (2006) 12(18):5543–9 10.1158/1078-0432.CCR-06-0632 17000691

[85] Ishida H Nakata T Sato N Li PK Kuwabara T Akinaga S Inhibition of steroid sulfatase activity and cell proliferation in ZR-75-1 and BT-474 human breast cancer cells by KW-2581 in vitro and in vivo. Breast Cancer Res Treat (2007) 104(2):211–9 10.1007/s10549-006-9404-8 17061037

[86] Ishida H Nakata T Suzuki M Shiotsu Y Tanaka H Sato N A novel steroidal selective steroid sulfatase inhibitor KW-2581 inhibits sulfated-estrogen dependent growth of breast cancer cells in vitro and in animal models. Breast Cancer Res Treat (2007) 106(2):215–27 10.1007/s10549-007-9495-x 17268815

[87] Rausch L Green C Steinmetz K LeValley S Catz P Zaveri N Preclinical pharmacokinetic, toxicological and biomarker evaluation of SR16157, a novel dual-acting steroid sulfatase inhibitor and selective estrogen receptor modulator. Cancer Chemother Pharmacol (2011) 67(6):1341–52 10.1007/s00280-010-1430-x 20737149

